# An Image-Guided Combination Strategy: Immediate Hepatic Arterial Infusion of Nivolumab Following Transarterial Chemoembolization for Unresectable Hepatocellular Carcinoma

**DOI:** 10.3390/cancers18060978

**Published:** 2026-03-18

**Authors:** Sujing Zhang, Zheng Zheng, Changwang Zhang, Xueqian Liu, Xinlei Shi, Wenhua Ma

**Affiliations:** Department of Oncology, The First Hospital of Hebei Medical University, No. 89 Donggang Road, Yuhua District, Shijiazhuang 050031, China

**Keywords:** hepatocellular carcinoma, transarterial chemoembolization, hepatic arterial infusion, nivolumab, propensity score matching

## Abstract

**Background:** Transarterial chemoembolization (TACE) is an established image-guided, minimally invasive therapy for unresectable hepatocellular carcinoma (HCC). However, post-embolization hypoxia often triggers compensatory angiogenesis and an immunosuppressive microenvironment, limiting long-term efficacy. We hypothesized that the immediate image-guided hepatic arterial infusion (HAI) of a PD-1 inhibitor following TACE could synergistically enhance local tumor control. **Methods:** In this retrospective, propensity-score-matched study, 226 patients with unresectable HCC (January 2021–June 2024) were analyzed. After 1:1 matching, 84 pairs were included: Study Group (TACE + HAI-nivolumab) and Control Group (TACE alone). Nivolumab (3 mg/kg) was infused via the hepatic artery under fluoroscopic guidance immediately after embolization. Primary endpoints were overall survival (OS) and progression-free survival (PFS); secondary endpoints included objective response rate (ORR) by mRECIST and changes in serum angiogenesis/immune biomarkers. **Results:** The Study Group demonstrated significantly longer median OS (16.2 vs. 12.8 months; HR 0.62, 95% CI: 0.44–0.88, *p* = 0.007) and median PFS (9.8 vs. 6.5 months; *p* < 0.001). ORR was higher with combination therapy (58.3% vs. 36.9%, *p* = 0.006). Mechanistically, HAI-nivolumab suppressed the post-TACE surge in VEGF and Ang-2 (*p* < 0.001) and increased the peripheral CD4+/CD8+ T-cell ratio. Grade 3/4 adverse events were comparable between groups (14.3% vs. 10.7%, *p* = 0.485). **Conclusions:** The image-guided combination of TACE with immediate HAI of nivolumab is associated with improved survival and tumor response in unresectable HCC. This strategy may counteract the adverse post-embolization microenvironment by simultaneously inhibiting angiogenesis and reactivating local immunity, representing an advanced image-guided combination therapy with strong translational relevance.

## Simple Summary

Primary liver cancer is a leading cause of cancer-related death, often treated with transarterial chemoembolization (TACE), a procedure that cuts off the tumor’s blood supply. However, the resulting lack of oxygen can paradoxically trigger new blood vessel growth and suppress the immune system, causing the tumor to return. This study investigated a novel strategy: infusing an immunotherapy drug, nivolumab, directly into the hepatic artery immediately after the standard procedure. The authors aimed to create a localized “immune shock” to counteract the tumor’s defensive mechanisms. The results indicate that this combination may extend patient survival and improve tumor shrinkage compared to standard treatment alone. By effectively stopping abnormal blood vessel formation and reactivating the body’s immune response, this image-guided approach provides a promising new therapeutic strategy for patients with inoperable liver tumors.

## 1. Introduction

Primary liver cancer remains a formidable global health challenge, with Hepatocellular Carcinoma (HCC) accounting for approximately 90% of cases [1,2]. It is the most common primary liver neoplasm [3] and ranks as the second leading cause of cancer-related mortality worldwide, with a particularly high prevalence in East Asia [4]. Despite the implementation of surveillance programs for high-risk populations, the insidious onset of HCC means that a significant proportion of patients are diagnosed at intermediate or advanced stages. For instance, in a contemporary cohort, patients with Barcelona Clinic Liver Cancer (BCLC) intermediate stage (B) and advanced stage (C) comprised 16.5% and 10.9% of cases, respectively [5,6,7], while another study focusing on intermediate-stage HCC reported that 37.1% of patients were in this category at diagnosis [8]. For these patients, curative interventions such as surgical resection or liver transplantation are often not feasible due to multifocal disease or vascular invasion. Consequently, locoregional and systemic therapies represent the mainstay of treatment.

Transarterial Chemoembolization (TACE) is the globally accepted standard of care for intermediate-stage HCC and is widely used for selected advanced cases [1]. The procedure involves the intra-arterial delivery of chemotherapeutic agents followed by embolization of the tumor-feeding vessels. Mechanistically, TACE induces extensive tumor necrosis through a dual hit of cytotoxicity and ischemia. However, TACE is rarely curative. The profound hypoxia induced by embolization creates a “hypoxic stress” microenvironment that paradoxically stabilizes hypoxia-inducible factor 1-alpha (HIF-1α). This leads to the transcriptional upregulation of vascular endothelial growth factor (VEGF), angiopoietin-2 (Ang-2), and other pro-angiogenic cytokines, which fuel neovascularization and promote residual tumor survival and metastasis [9,10].

Parallel to angiogenic activation, the immune landscape of HCC plays a pivotal role in treatment failure. While TACE can induce immunogenic cell death (ICD) and release tumor-associated antigens, the liver microenvironment is inherently immunosuppressive (tolerogenic). This environment is characterized by T-cell exhaustion, the accumulation of regulatory T cells (Tregs), and the upregulation of immune checkpoints such as PD-L1 on tumor and stromal cells, which are key mechanisms of immune escape in HCC [11]. This immunosuppressive network often dampens the host’s antitumor immune response triggered by TACE. Although systemic immune checkpoint inhibitors (ICIs) like nivolumab and pembrolizumab have revolutionized HCC management and are established treatment options [12], single-agent objective response rates remain modest at −15% [13], suggesting that systemic administration alone may not be sufficient to overcome the dense, immunosuppressive stromal barrier of liver tumors.

Recent therapeutic strategies have focused on combination approaches. Theoretical models and preclinical studies suggest that combining TACE with immunotherapy could be synergistic: TACE can serve as an in situ vaccine platform by releasing tumor antigens and inducing immunogenic cell death, while immune checkpoint inhibitors (ICIs) help to prevent the exhaustion of the newly primed T cells and sustain anti-tumor immunity [14]. However, a critical limitation of systemic ICI administration is the potential for immune-related adverse events (irAEs) and insufficient drug concentration within the tumor core. Hepatic Arterial Infusion (HAI) offers a unique pharmacological advantage by delivering therapeutic agents directly into the hepatic artery, the primary blood supply for HCC. This route achieves a high local drug concentration with a significant “first-pass” effect, maximizing exposure to the tumor while minimizing systemic dilution and toxicity [15]. While HAI of chemotherapeutic agents is well-established, robust clinical data on the safety and efficacy of HAI for delivering PD-1 inhibitors remains sparse, representing a novel investigational frontier, though early combination regimens show promise [16].

**Figure 1:**
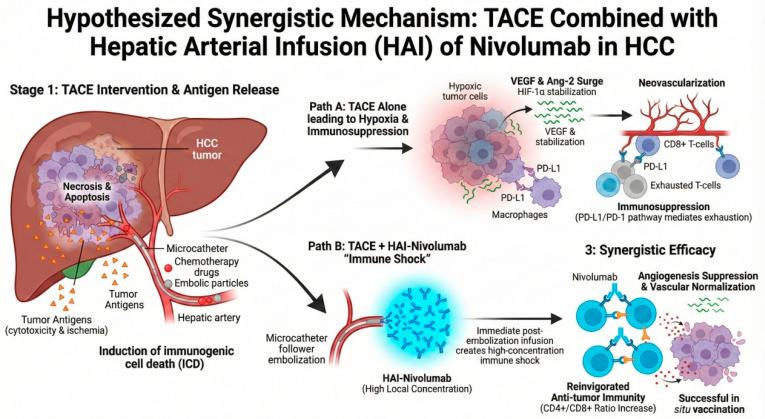
Hypothesized synergistic mechanism of TACE combined with hepatic arterial infusion (HAI) of nivolumab. Transarterial chemoembolization (TACE) induces extensive tumor necrosis and ischemia, subsequently releasing tumor-associated antigens into the microenvironment. However, the resulting hypoxia paradoxically upregulates angiogenic factors, such as VEGF and Ang-2, promoting neovascularization. Simultaneously, the post-TACE environment is characterized by immunosuppression, including T-cell exhaustion and upregulation of PD-L1, which dampens the host antitumor response. The proposed strategy involves the immediate hepatic arterial infusion (HAI) of nivolumab following embolization to achieve a high local drug concentration (“immune shock”). This approach is hypothesized to synergistically overcome therapeutic resistance by blocking PD-1-mediated T-cell exhaustion, reinvigorating antigen-primed cytotoxic T-cells to act as an in situ vaccine, and concurrently suppressing hypoxia-driven angiogenesis.

In this study, we hypothesized that administering nivolumab via the hepatic artery immediately post-embolization would create a “high-concentration immune shock” that synergistically counteracts the post-TACE angiogenic surge and reinvigorates local immunity (Figure 1). To test this hypothesis while mitigating the biases inherent in observational studies, we conducted a propensity score-matched (PSM) retrospective analysis to evaluate the real-world efficacy, safety, and biomarker dynamics of TACE combined with HAI-nivolumab versus TACE alone in patients with unresectable HCC.

## 2. Materials and Methods

### 2.1. Study Design and Ethical Oversight

This single-center, retrospective cohort study was conducted at The First Hospital of Hebei Medical University. We comprehensively reviewed the electronic medical records and radiological data of patients diagnosed with unresectable HCC who underwent interventional therapy between January 2021 and June 2024. The study was conducted in accordance with the Declaration of Helsinki, and approved by the Institutional Ethics Committee of The First Hospital of Hebei Medical University (Protocol code: 20220171; Date of approval: 15 March 2020). Given the retrospective nature of the analysis, the requirement for written informed consent for data collection was waived; however, all patients had provided written informed consent for the off-label use of HAI-nivolumab and TACE procedures at the time of treatment.

### 2.2. Patient Population and Eligibility Criteria

A total of 226 patients were initially screened. The inclusion criteria were defined as follows: (1) Confirmed diagnosis of HCC based on the American Association for the Study of Liver Diseases (AASLD) guidelines or histological confirmation via biopsy; (2) Unresectable status, defined as BCLC stage B (intermediate) or stage C (advanced) with limited extrahepatic spread, or stage A patients who were medically unfit for or refused surgery/ablation; (3) Child–Pugh liver function class A or B (score ≤7); (4) Eastern Cooperative Oncology Group (ECOG) performance status of 0 or 1; and (5) Presence of at least one measurable lesion according to Modified Response Evaluation Criteria in Solid Tumors (mRECIST) [17]. Patients were excluded if they met any of the following criteria: (1) Presence of complete portal vein thrombosis (Vp4) involving the main trunk; (2) Prior exposure to systemic therapy (TKIs) or other immunotherapy agents; (3) Active autoimmune disease requiring systemic immunosuppression; (4) History of other synchronous or metachronous malignancies within 5 years; or (5) Incomplete clinical or follow-up data.

### 2.3. Treatment Protocols

**Transarterial Chemoembolization (TACE):** All patients underwent conventional TACE (cTACE) performed by interventional radiologists with over 10 years of experience. While Drug-Eluting Bead TACE (DEB-TACE) is also a valid option [18], cTACE using Lipiodol was chosen as the standard at our institution to allow better visualization of the tumor bed during subsequent HAI. The Seldinger technique was used to access the femoral artery. A 5-Fr catheter was introduced, and angiography was performed to map the hepatic arterial anatomy. A microcatheter (Progreat, Terumo, Japan) was then advanced superselectively into the tumor-feeding arteries. An emulsion was prepared by mixing Lipiodol (5–20 mL) with chemotherapeutic agents (typically Epirubicin 30–50 mg and/or Lobaplatin 30–50 mg). This emulsion was infused into the tumor, followed by embolization with gelatin sponge particles until blood flow stasis was achieved.

**Study Group (TACE + HAI-Nivolumab):** Patients in this group received a novel combination regimen. Immediately following the embolization phase, while the microcatheter was still positioned in the proper hepatic artery (or the dominant feeding artery for bilobar disease), Nivolumab (Opdivo, BMS) was administered. The rationale for this timing was to target the immediate hypoxic surge and utilize the “vascular lock” effect of embolization to potentially retain the immunotherapeutic agent. The dose was 3 mg/kg diluted in 100 mL of 0.9% normal saline, infused via an infusion pump over a period of 30 min. HAI-nivolumab was administered only during the first TACE session to serve as an induction therapy. The rationale was to bathe the residual tumor microenvironment in a high concentration of PD-1 inhibitor immediately after the ischemic insult. Following discharge, these patients received maintenance intravenous (IV) nivolumab (3 mg/kg) every 3 weeks until disease progression or unacceptable toxicity.

**Control Group (TACE Alone):** Patients in the Control Group received the standard TACE procedure described above without the intra-arterial infusion of nivolumab. TACE was repeated “on-demand” in both groups based on follow-up imaging (CT/MRI) and liver function, typically at intervals of 6–8 weeks. Subsequent TACE procedures were standard cTACE in both groups.

### 2.4. Outcomes and Assessments

**Survival Endpoints:** The primary endpoints were Overall Survival (OS), defined as the time from the first TACE procedure to death from any cause, and Progression-Free Survival (PFS), defined as the time to radiological progression per mRECIST or death. Patients were followed up until 31 December 2025.

**Tumor Response and Safety:** Secondary endpoints included the Objective Response Rate (ORR), defined as the percentage of patients achieving Complete Response (CR) or Partial Response (PR), and the Disease Control Rate (DCR). Tumor response was assessed by two independent radiologists using mRECIST criteria [17] at 1–3 months post-treatment. Adverse events (AEs) were graded according to the Common Terminology Criteria for Adverse Events (CTCAE) version 5.0 [19].

**Biomarker Analysis:** To investigate the underlying mechanism, peripheral blood samples were collected at baseline (within 3 days prior to TACE) and 1 month after the first treatment. Serum levels of angiogenic factors—VEGF, VEGFR-2, and Ang-2—were quantified using enzyme-linked immunosorbent assay (ELISA) kits. Additionally, immune cell subsets (CD3+, CD4+, CD8+) were analyzed via flow cytometry to calculate the CD4+/CD8+ ratio, serving as a surrogate marker for systemic immune activation.

### 2.5. Statistical Analysis and Propensity Score Matching

To minimize the selection bias inherent in non-randomized observational studies, we employed Propensity Score Matching (PSM). A logistic regression model was constructed to calculate the propensity score for each patient based on the following covariates: age, gender, hepatitis B virus (HBV) status, BCLC stage, tumor number, maximum tumor size, and baseline alpha-fetoprotein (AFP) level. Patients in the Study Group were matched 1:1 with those in the Control Group using the nearest-neighbor method with a caliper width of 0.02.

Continuous variables were expressed as mean ± standard deviation (SD) and compared using Student’s *t*-test or Mann–Whitney U test. Categorical variables were presented as frequencies (percentages) and compared using Chi-square or Fisher’s exact tests. Survival curves were estimated using the Kaplan–Meier method and compared via the log-rank test. Cox proportional hazards regression models were used to calculate Hazard Ratios (HR) and 95% Confidence Intervals (CI). Potential multicollinearity among the covariates included in the logistic regression model was assessed using the Variance Inflation Factor (VIF). All selected variables demonstrated a VIF <5, indicating no severe multicollinearity. All statistical analyses were performed using SPSS software (v27.0) and R (v4.3.0). A two-sided *p*-value < 0.05 was considered statistically significant.

## 3. Results

### 3.1. Baseline Characteristics and PSM

**Figure 2:**
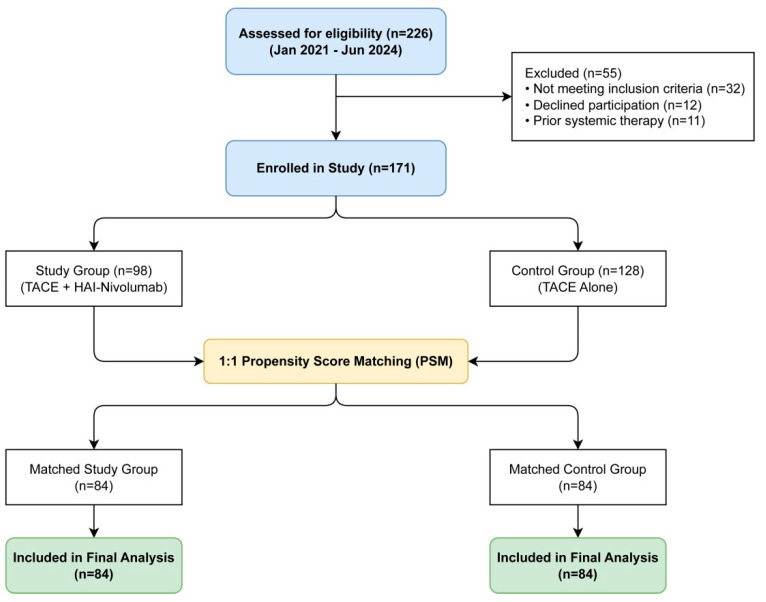
Study Flowchart. The diagram illustrates the patient selection process. Of 226 patients assessed, 55 were excluded due to inclusion/exclusion criteria. The remaining 173 patients were matched 1:1 using Propensity Score Matching (PSM), resulting in 84 patients in the Study Group (TACE + HAI-–Nivolumab) and 84 in the Control Group (TACE Alone) for the final analysis. Baseline characteristics were balanced between groups after matching (see Table 1 and Table S1 for pre-matching data).

A total of 226 eligible patients were initially identified (Study Group, *n* = 98; Control Group, *n* = 128). The patient selection process is detailed in Figure 2. Baseline characteristics before matching are shown in Table 1. Before matching, the Study Group had a slightly higher proportion of BCLC C patients. After 1:1 PSM, 84 matched pairs were generated. Baseline characteristics including age, tumor burden, and liver function were well-balanced between the two groups (*p* > 0.05) as shown in Table 1. Notably, the presence of liver cirrhosis was comparable between groups (81.0% vs. 83.3%, *p* = 0.688), and tumor number was also balanced.

### 3.2. Tumor Response and Survival Analysis

At the data cutoff date of 31 December 2025, the median follow-up was 18.6 months. The Study Group achieved a significantly higher Objective Response Rate (ORR) of 58.3% compared to 36.9% in the Control Group (*p* = 0.006). The Disease Control Rate (DCR) was also improved (89.3% vs. 71.4%, *p* = 0.004) (Table 2). Notably, 5 patients (6.0%) in the Study Group and 2 (2.4%) in the Control Group were successfully downstaged (meeting the criteria for curative resection or liver transplantation) and underwent subsequent salvage surgery or ablation.

**Figure 3:**
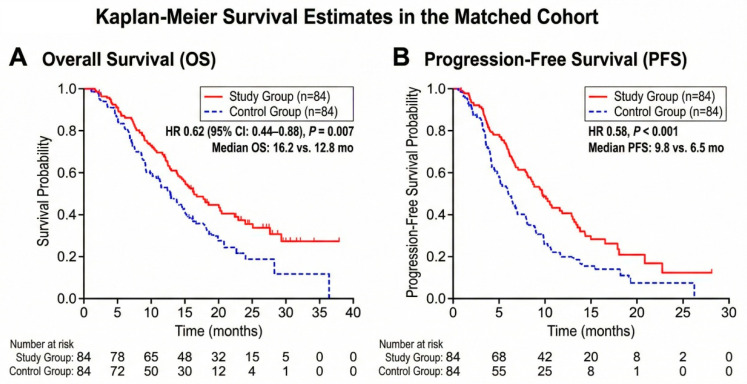
Kaplan–Meier Survival Estimates in the Matched Cohort. (**A**) Overall Survival (OS). The Study Group (red line) showed significantly longer median OS compared to the Control Group (blue dashed line) (16.2 vs. 12.8 months, *p* = 0.007). (**B**) Progression-Free Survival (PFS). The Study Group demonstrated superior PFS (9.8 vs. 6.5 months, *p* < 0.001).

Kaplan–Meier analysis revealed a substantial survival benefit for the combination therapy. The median Overall Survival (OS) was 16.2 months (95% CI: 14.1–18.3) in the Study Group versus 12.8 months (95% CI: 11.2–14.4) in the Control Group (HR 0.62, 95% CI: 0.44–0.88, *p* = 0.007) (Figure 3A). Similarly, the median Progression-Free Survival (PFS) was extended from 6.5 months to 9.8 months (HR 0.58, *p* < 0.001) (Figure 3B).

### 3.3. Mechanism: Angiogenesis Suppression and Immune Activation

**Figure 4:**
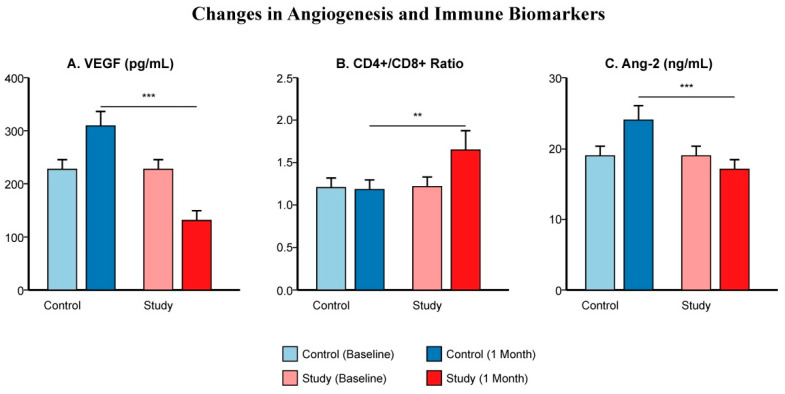
Changes in Angiogenesis and Immune Biomarkers. Bar charts comparing baseline and 1-month post-treatment levels with error bars representing standard deviation. (**A**) Serum VEGF levels surged in the Control Group but were suppressed in the Study Group (*p* < 0.001). (**B**) The peripheral CD4+/CD8+ T-cell ratio significantly increased in the Study Group, indicating systemic immune activation. (**C**) Ang-2 levels followed a trend similar to VEGF. Note: These biomarkers serve as surrogate indicators measured in peripheral blood. ***, *p* < 0.001; **, *p* < 0.01.

To elucidate the biological mechanism, we analyzed serum biomarkers. In the Control Group, TACE induced a significant surge in VEGF levels one month post-treatment compared to baseline. Strikingly, this angiogenic surge was effectively abrogated in the Study Group (*p* < 0.001) (Figure 4A), with similar suppression observed for Ang-2 (Figure 4C). Furthermore, the Study Group exhibited a significant increase in the peripheral CD4+/CD8+ T-cell ratio (Figure 4B), indicating a shift towards an active immune phenotype, whereas no such change was observed in the Control Group.

### 3.4. Safety

The combination regimen was well-tolerated. The incidence of Grade 3/4 adverse events was comparable between the two groups (14.3% vs. 10.7%, *p* = 0.485) (Table 2). Although immune-related adverse events (irAEs) such as rash and hypothyroidism were more frequent in the Study Group, they were predominantly Grade 1–2 and manageable with standard supportive care. No treatment-related mortality occurred.

## 4. Discussion

This study provides compelling real-world evidence that the combination of TACE and HAI-nivolumab significantly improves survival outcomes and tumor responses in patients with unresectable HCC. By rigorously utilizing propensity score matching, we mitigated selection bias and demonstrated that this novel locoregional immunomodulatory strategy is associated with a 38% reduction in the risk of death (HR 0.62) compared to TACE alone. These findings underscore the potential of route-specific immunotherapy administration in enhancing therapeutic efficacy for intermediate-stage malignancies.

Our results align with and significantly extend the findings of recent pivotal trials. While the IMbrave150 [20] and LEAP-002 [21] trials have established the dominant role of systemic combination therapies (ICI + anti-VEGF or TKI), our study highlights the unique and often overlooked value of the intra-arterial route for delivering immunotherapeutics. The pharmacokinetics of HAI allow for an extremely high local drug concentration, which is crucial for overcoming the “cold” or immune-excluded microenvironment typical of liver tumors by mechanisms such as inducing immunogenic cell death and reshaping the local immune landscape [22,23,24]. The observed Objective Response Rate (ORR) of 58.3% in our study is numerically superior to historical controls for TACE alone (−30–40%) [25,26,27] and is comparable to the aggressive triplet therapies (TACE plus lenvatinib and pembrolizumab) reported in the recent LEAP-012 trial [28]. This high response rate suggests that the initial “immune shock” delivered via HAI may lower the threshold for tumor antigen recognition and T-cell activation.

A key mechanistic insight from our study is the confirmation of a “vascular normalization” effect associated with the combination therapy. It is well-documented that hypoxia induced by TACE is a double-edged sword: while it causes tumor necrosis, it also inevitably upregulates HIF-1α, leading to a compensatory surge in VEGF that fuels rapid revascularization and recurrence [29,30,31]. Our biomarker analysis (Figure 3) confirmed that the addition of HAI-nivolumab significantly blunted this post-TACE VEGF surge. This supports the emerging concept that activated CD8+ T cells can secrete interferon-gamma (IFN-γ), which acts on tumor stroma to downregulate angiogenesis and promote vascular normalization [18,32,33]. Thus, the immunotherapy not only attacks the tumor cells directly but also remodels the vascular niche, potentially making the remaining tumor more accessible to subsequent treatments. However, it is important to note that these biomarkers were measured in peripheral blood, serving as surrogate indicators. Direct intratumoral evidence was not available due to the lack of serial biopsies.

Furthermore, the concurrent increase in the peripheral CD4+/CD8+ ratio in the Study Group indicates that the local administration of nivolumab successfully reinvigorated systemic immunity. TACE is known to release a plethora of tumor antigens, which can promote antitumor immunity [34,35,36]; however, without checkpoint inhibition, these antigens often lead to abortive T-cell activation or exhaustion. By providing high-dose PD-1 blockade at the precise moment of antigen release (post-embolization), we hypothesize that HAI-nivolumab prevents this exhaustion, converting the TACE event into a successful in situ vaccination. This concept is supported by preclinical and clinical rationale indicating TACE can modulate the tumor microenvironment by increasing the CD4+/CD8+ ratio, suggesting a positive interaction with immune checkpoint inhibitor activity [37,38]. Encouraging efficacy of combined TACE and PD-1 inhibition has been observed in clinical settings, including studies evaluating TACE combined with hepatic arterial infusion of nivolumab, as well as in clinical trials and real-world analyses of PD-1 inhibitors combined with TACE for unresectable hepatocellular carcinoma [39,40].

Regarding safety, a primary concern with HAI immunotherapy has been the potential for inducing severe immune-mediated hepatitis due to high local drug exposure. Encouragingly, our data did not support this fear. The rates of grade 3/4 hepatic toxicity were similar between the Study and Control groups (14.3% vs. 10.7%, *p* = 0.485), consistent with preliminary findings from the phase II IMMUTACE trial [41]. This suggests that the liver parenchyma tolerates high local concentrations of PD-1 inhibitors remarkably well, likely due to the rapid binding of the antibody to the high tumor antigen load and the high density of PD-1+ immune cells within the tumor, acting as a “sink” that protects the healthy liver tissue.

Despite these promising results, our study has limitations. First, despite the rigorous use of PSM to balance baseline characteristics, the retrospective nature of the study inherently carries the risk of selection bias and unmeasured confounding factors, such as liver stiffness. Second, as a single-center study, the technical expertise in HAI administration may vary compared to other centers. Third, a specific limitation is the difficulty in isolating the therapeutic contribution of the initial HAI route versus the subsequent systemic maintenance nivolumab. The survival benefit may reflect the combination of early high-dose local priming and sustained systemic checkpoint inhibition. Finally, TACE techniques have evolved; while our study utilized cTACE, Drug-Eluting Bead TACE (DEB-TACE) has also demonstrated efficacy and safety, as highlighted by Spreafico et al. [42], offering consistent drug delivery. Future randomized trials should address these variables.

## 5. Conclusions

By inhibiting hypoxia-driven angiogenesis and reprogramming the immune microenvironment, this regimen may offer a survival benefit with a manageable safety profile. This combination strategy may be a valuable addition to the interventional oncology armamentarium for unresectable HCC.

## Figures and Tables

**Table 1:** Baseline Characteristics of Patients in the Propensity Score-Matched Cohort (*n* = 168).

Characteristic	Study Group (*n* = 84)	Control Group (*n* = 84)	*p*-Value
Age, years (mean ± SD)	59.8 ± 10.2	60.5 ± 9.8	0.645
Gender (Male), *n* (%)	68 (81.0%)	70 (83.3%)	0.688
Cirrhosis present, *n* (%)	68 (81.0%)	70 (83.3%)	0.688
HBV Infection, *n* (%)	70 (83.3%)	69 (82.1%)	0.836
BCLC Stage, *n* (%)			0.814
- Stage B	34 (40.5%)	36 (42.9%)	
- Stage C	50 (59.5%)	48 (57.1%)	
Max Tumor Size, cm	7.8 ± 2.4	7.6 ± 2.2	0.572
Tumor number > 3, *n* (%)	40 (47.6%)	38 (45.2%)	0.757
TACE sessions, mean ± SD	2.4 ± 1.1	2.3 ± 1.2	0.575
AFP > 400 ng/mL, *n* (%)	45 (53.6%)	42 (50.0%)	0.643

**Table 2:** Tumor Response (mRECIST) and Adverse Events.

Outcome	Study Group (*n* = 84)	Control Group (*n* = 84)	*p*-Value
**Tumor Response (Best Response)**			
Complete Response (CR)	15 (17.9%)	6 (7.1%)	0.034
Partial Response (PR)	34 (40.5%)	25 (29.8%)	0.147
Stable Disease (SD)	26 (31.0%)	29 (34.5%)	0.621
Progressive Disease (PD)	9 (10.7%)	24 (28.6%)	0.003
**ORR (CR + PR)**	49 (58.3%)	31 (36.9%)	0.006
**Safety Profile (Grade 3/4 Events)**			
Liver Enzyme Elevation (AST/ALT)	6 (7.1%)	5 (6.0%)	0.754
Hyperbilirubinemia	3 (3.6%)	2 (2.4%)	0.650
Rash (Immune-related)	2 (2.4%)	0 (0%)	0.154
**Total Grade 3/4 Events**	12 (14.3%)	9 (10.7%)	0.485

## Data Availability

The datasets used and/or analysed during the current study are available from the corresponding author on reasonable request.

## Supplementary Materials

The following supporting information can be downloaded at: https://www.mdpi.com/article/10.3390/cancers18060978/s1. Table S1: Baseline Characteristics of Patients Before Propensity Score Matching (*n* = 226).

## Abbreviations

HCC	Hepatocellular Carcinoma
TACE	Transarterial Chemoembolization
HAI	Hepatic Arterial Infusion
ICI	Immune Checkpoint Inhibitor
PD-1	Programmed Death-1
PD-L1	Programmed Death-Ligand 1
VEGF	Vascular Endothelial Growth Factor
Ang-2	Angiopoietin-2
HIF-1α	Hypoxia-Inducible Factor 1-alpha
BCLC	Barcelona Clinic Liver Cancer
ECOG	Eastern Cooperative Oncology Group
mRECIST	Modified Response Evaluation Criteria in Solid Tumors
OS	Overall Survival
PFS	Progression-Free Survival
ORR	Objective Response Rate
DCR	Disease Control Rate
PSM	Propensity Score Matching
HR	Hazard Ratio
CI	Confidence Interval
AE	Adverse Event
irAE	Immune-related Adverse Event
ICD	Immunogenic Cell Death
