# Supplementary material for: An Image-Guided Combination Strategy: Immediate Hepatic Arterial Infusion of Nivolumab Following Transarterial Chemoembolization for Unresectable Hepatocellular Carcinoma
Source: Cancers (Basel). 2026 Mar 18;18(6):978. doi: 10.3390/cancers18060978 (PMC13025836; doi:10.3390/cancers18060978)
Supplement: Supplementary file 1 [file cancers-18-00978-s001.zip › cancers-4141123-supplementary.pdf]

Supplementary Table S1. Baseline Characteristics of Patients Before Propensity Score Matching (n=226).

Characteristic	Study Group (n=98)	Control Group (n=128)	*P*-value
Age, years (mean ± SD)	59.5 ± 10.5	61.2 ± 10.1	0.224
Gender (Male), n (%)	78 (79.6%)	105 (82.0%)	0.638
Cirrhosis present, n (%)	80 (81.6%)	102 (79.7%)	0.715
HBV Infection, n (%)	81 (82.7%)	104 (81.3%)	0.791
BCLC Stage, n (%)			0.032*
- Stage B	35 (35.7%)	64 (50.0%)	
- Stage C	63 (64.3%)	64 (50.0%)	
Max Tumor Size, cm (mean ± SD)	8.1 ± 2.6	7.4 ± 2.3	0.038*
Tumor number >3, n (%)	52 (53.1%)	54 (42.2%)	0.104
TACE sessions, mean ± SD	2.5 ± 1.1	2.2 ± 1.0	0.035*
AFP > 400 ng/mL, n (%)	55 (56.1%)	60 (46.9%)	0.168

Abbreviations: SD, standard deviation; HBV, hepatitis B virus; BCLC, Barcelona Clinic Liver Cancer; TACE, transarterial chemoembolization; AFP, alpha-fetoprotein.

* Statistically significant ($P < 0.05$). Note that before matching, the Study Group had a significantly higher proportion of BCLC stage C patients, larger maximum tumor sizes, and a slightly higher number of TACE sessions compared to the Control Group. These imbalances were successfully eliminated after PSM (see Table 1).
